# Hypoosmolar Hyponatremia as Presenting Symptom of a Rare Case of Olfactory Neuroblastoma

**DOI:** 10.1210/jcemcr/luaf130

**Published:** 2025-06-23

**Authors:** Sarah Rieder, Sabina Brigitte Streuli, Grischa Marti, Urs Borner, Patrick Kempf

**Affiliations:** Medical Department, Thun Hospital, Thun 3600, Switzerland; Medical Department, Thun Hospital, Thun 3600, Switzerland; Medical Department, Thun Hospital, Thun 3600, Switzerland; Department of Otorhinolaryngology, Head and Neck Surgery, Inselspital, Bern University Hospital, University of Bern, Bern 3010, Switzerland; Medical Department, Thun Hospital, Thun 3600, Switzerland

**Keywords:** SIAD, olfactory neuroblastoma, esthesioneuroblastoma, hyponatremia

## Abstract

Hyponatremia is a common electrolyte disturbance. The syndrome of inappropriate antidiuresis (SIAD), characterized by nonosmotic release of arginine vasopressin, is a typical cause of hyponatremia. Ectopic secretion of arginine vasopressin by a tumor is often associated with small cell lung cancer but can also be seen in other neoplasms. We present a rare case of a previously healthy 34-year-old woman, evaluated for refractory hypoosmolar hyponatremia consistent with SIAD. After excluding other causes of SIAD, imaging revealed contrast-enhancing mucosal thickening in the left maxillary sinus with expansion into the sphenoid and frontal sinuses, leading to the diagnosis of olfactory neuroblastoma. After surgical removal, hyponatremia resolved. Although treatment of olfactory neuroblastoma typically involves surgery and radiotherapy, there are no standardized therapeutic guidelines, due to the low prevalence of the disease. In summary, olfactory neuroblastoma is an extremely rare paraneoplastic cause of SIAD that should not be overlooked after excluding other possible etiologies.

## Introduction

Hyponatremia is a common electrolyte disorder affecting approximately 5% of adults and up to 35% of hospitalized patients [1-3]. Symptoms are primarily neurologic and vary depending on severity and acuteness of onset. They may include hyporeflexia, nausea, vertigo, headaches, seizures, somnolence, and Cushing reflex with bradycardia, hypertension, and abnormal respirations [1-3].

Hypotonic hyponatremia is frequently caused by the syndrome of inappropriate antidiuresis (SIAD), defined by nonosmotic release of arginine vasopressin (antidiuretic hormone) [2, 3].

SIAD can be idiopathic or secondary to various underlying conditions, such as central nervous system pathologies, drugs, pulmonary diseases, infections, surgery, hereditary disorders, or malignancies, whereas hypocortisolism and severe hypothyroidism can mimic SIAD [2, 3].

Paraneoplastic SIAD is most often due to small cell lung cancer (SCLC) but can also be caused by rare malignancies such as olfactory neuroblastoma [4].

Olfactory neuroblastoma (ONB), also known as *esthesioneuroblastoma*, is a rare malignant tumor of the nasal cavity, typically originating from specialized sensory cells of the olfactory epithelium [5].

Symptoms of ONB are often nonspecific and usually related to the mass effect of the tumor, for example, nasal obstruction, epistaxis or olfactory disorders [6].

Paraneoplastic syndromes including SIAD are rarely described [4]. Because of its nonspecific symptoms and rarity, management of ONB remains difficult [4, 6].

We present a case of a young patient with ONB, who initially presented with hypotonic hyponatremia.

## Case Presentation

A previously healthy 34-year-old woman was referred for idiopathic hyponatremia of 125 mEq/L (SI: 125 mmol/L) (reference range, 136-145 mEq/L [SI: 136-125 mmol/L]). She reported slow-progressing fatigue over several weeks and frequent headaches. No nasal symptoms were noted. The patient's medical history included migraines, an appendectomy several years prior, varicosis, and the birth of a child a year prior. There was no history of cardiac, renal, or hepatic disease, central nervous system trauma, or surgery. Menstrual cycles were regular every 4 weeks. A random serum sodium had been normal 4 years earlier.

There was no obvious pharmacological cause of hyponatremia. Documented medications included a hormonal intrauterine device and sodium chloride tablets, initiated weeks prior for hyponatremia management.

Her family history was significant for differentiated thyroid carcinoma. Fluid restriction and oral sodium chloride substitution had brought only slight improvement in sodium levels.

## Diagnostic Assessment

Initial evaluation confirmed hypoosmolar hyponatremia with a sodium level of 123 mEq/L (SI: 123 mmol/L) and serum osmolality of 256 mOsm/kg (SI: 256 mmol/kg) (reference range, 280-300 mOsm/kg [SI: 280-300 mmol/kg]). The patient was euvolemic and frequent causes of pseudohyponatremia were excluded. Subsequent measurements confirmed hyponatremia with a nadir of 118 mEq/L (SI: 118 mmol/L). Diagnosis of SIAD was established based on an inadequately concentrated urine (urine sodium 77 mEq/L [SI: 77 mmol/L] [reference range, 26.7-146.7 mEq/L; SI: 26.7-146.7 mmol/L], urine osmolality 966 mOsm/kg [SI: 966 mmol/kg] [reference range, 50-1500 mOsm/kg; SI: 50-1500 mmol/kg]).

Hypocortisolism was excluded with a fasting serum cortisol level of 15.58 µg/dL (SI: 430 nmol/L) (reference range, 5.25-22.43 µg/dL [SI: 145-619 nmol/L]) [7], and the patient was euthyroid. Copeptin was increased at 11.16 pg/mL (SI: 41 pmol/L) (reference range, < 7.68 pg/mL [SI: < 28.2 pmol/L]).

A human immunodeficiency virus (HIV) test as well as a basic drug screening were negative. Physical examination and inflammatory parameters (whole blood count, C-reactive protein) showed no evidence of infection. Furthermore, a history of eunatremia several years prior rendered a genetic cause unlikely.

**Figure 1.**
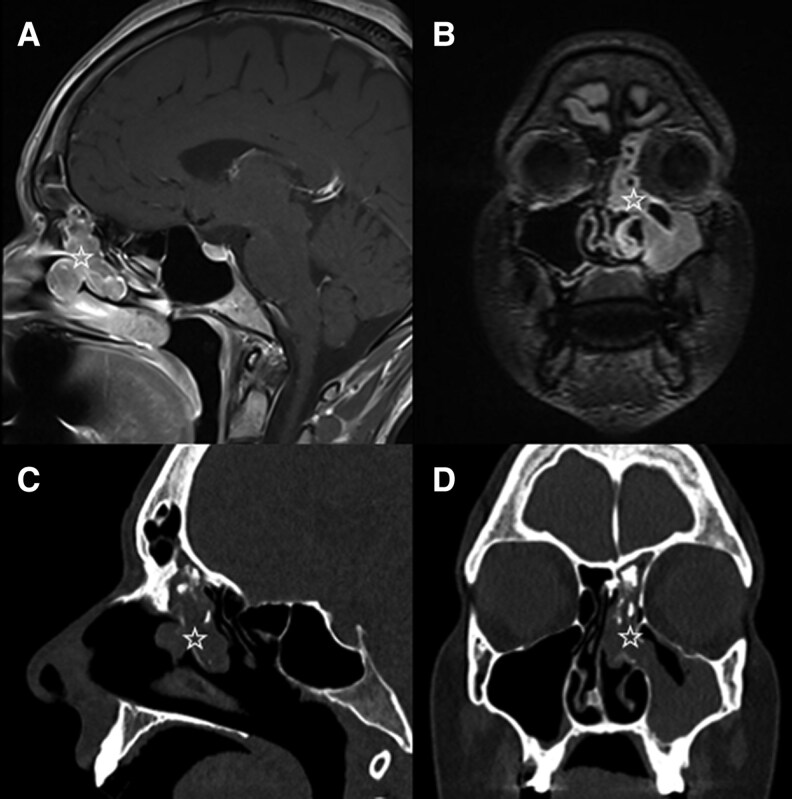
The T2-weighted sequence of a sagittal (A) and coronar (B) post-contrast MRI demonstrates a contrast-enhancing mass in the left maxillary sinus, extending into the sphenoid and frontal sinuses, with diffuse swelling of the middle and superior nasal conchae (star). A sagittal (C) and coronar (D) non-contrast CT scan shows intrasinusal and intraductal expansion (star) with destructive bone alterations.

Magnetic resonance imaging (MRI) of the skull revealed a contrast-enhancing mass in the left maxillary sinus, extending into the sphenoid and frontal sinuses, with diffuse swelling of the middle and superior nasal conchae (Fig. 1A and 1B). A thyroid sonography showed no abnormalities. An MRI of the thorax and the abdomen showed no sign of infectious or neoplastic disease, especially no sign of SCLC or tuberculosis. Chromogranin A levels were normal (47 µg/L [SI: 0.959 nmol/L] [reference range, < 101.9 µg/L; SI: < 2.079 nmol/L]).

**Figure 2.**
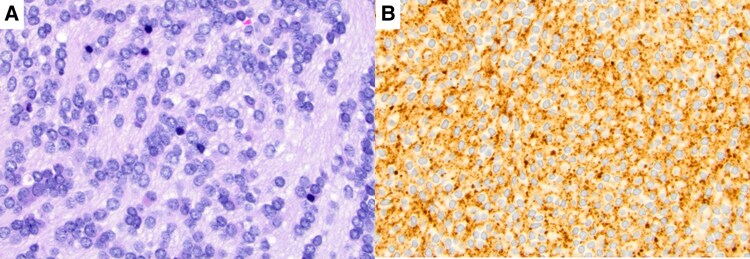
Histologic findings of the excisional biopsy of the left middle nasal meatus showing components of an olfactory neuroblastoma. Hematoxylin and eosin staining (A) shows typical tumor cells and a fibrillary matrix. In immunohistochemistry (B) tumor cells are positive for synaptophysin.

On further investigation, a nasal endoscopy revealed a pink-appearing tumor without telangiectasia in the middle nasal meatus. Excisional biopsy showed tumor cells with a basophilic nucleus and salt-and-pepper chromatin, poorly defined cytoplasm as well as the formation of Homer Wright rosettes embedded within abundant neuropil (Fig. 2A). In immunohistochemistry, the tumor cells were positive for synaptophysin (Fig. 2B) and exhibited a proliferative activity (Ki-67) of 1% to 2%, leading to diagnosis of an ONB, Hyams grade I [8, 9]. The diagnostic workup for staging and treatment planning was completed with a computerized tomography (CT) scan of the paranasal sinuses, which confirmed the tumor of the nasal cavity measuring approx. 30 mm × 21 mm × 19 mm (Fig. 1C and 1D). Furthermore, intrasinusal and intraductal (lacrimal duct) expansions, an accentuated left lacrimal gland, destructive bone alterations of the left maxillary sinus and erosion of the cribriform plate with possible intracerebral extension were present. A CT scan of the chest showed no intrathoracic tumor manifestation or lymph node involvement. Consequently, diagnosis of an ONB of the left nasal cavity and ethmoid, Hyams grade I [8], Kadish staging B [10, 11], pT1, cN0, cM0, was established.

## Treatment

Fluid restriction and oral substitution were continued, stabilizing hyponatremia between 125 and 130 mEq/L (SI: 125-130 mmol/L). Since surgical treatment was anticipated, additional therapies such as urea or vaptans were not administered. The patient underwent transnasal endoscopic tumor excision with computer assisted navigation, including complete left ethmoidectomy, maxillary sinusotomy, frontal sinusotomy, and resection of the middle nasal concha. Postoperative histological analysis revealed residual tumor cells, necessitating repeat endoscopy, re-ethmoidectomy, and mucosal resection. Adjuvant fractioned, highly conformal proton therapy was administered at a total dose of 66 Gy (relative biological effectiveness).

## Outcome and Follow-Up

Follow-up after the first surgery showed a complete remission of hyponatremia (serum sodium 137 mEq/L [SI: 137 mmol/L]) and a significant decrease in copeptin (0.84 pg/mL [SI: 3.1 pmol/L]). The patient was asymptomatic and fluid restriction was lifted.

## Discussion

This case highlights a rare instance of ONB presenting with paraneoplastic SIAD. Elevated copeptin despite severe hyponatremia suggested paraneoplastic, ectopic secretion of vasopressin (SIAD type A) [12].

ONB accounts for only 5% of intranasal tumors and 0.3% of all upper aerodigestive tract malignancies [6, 13]. ONB is a rare cause of hyponatremia that may remain undiagnosed for many years in patients with persistent SIAD of unknown origin, especially in younger patients [14].

Apart from SIAD, ONB can also cause ectopic ACTH secretion, hypercalcemia, hyperprolactinemia, catecholamine secretion, and neurologic paraneoplastic syndromes [4]. Tumor removal often leads to syndrome resolution [4], as illustrated in this case.

Imaging (CT and MRI) plays a central role in identifying ONB [6]. The classical finding is a “dumbbell-shaped” mass at the cribriform plate [15]. Cervical imaging is recommended as lymph node involvement occurs in up to 30% of ONB cases [13, 16]. Additionally, histological confirmation and grading via endoscopic biopsy is essential [6, 16].

Although ONB shares histological features with neuroendocrine tumors, its histologic maturation and differentiation vary significantly. This variability led to the development of the Hyams grading system, which assesses architectural and cytological markers. It distinguishes ONB into 4 groups ranging from well differentiated (grade I) to least differentiated (grade IV) [8].

A significant correlation between Hyams grades and the clinical behavior of ONB was shown [6, 13]. However, the Hyams system, unchanged since 1988, is based solely on morphology. Recent studies have suggested the Ki-67 proliferation index as an additional prognostic factor in ONB. Ki-67 index might be more reproducible than Hyams grading [9, 17].

A lower Ki-67 index was shown in Hyams grade I-II compared to Hyams grade III-IV [9]. This is consistent with the Ki-67 index of 1% to 2% observed in this case. A high Ki-67 index, especially exceeding 25%, is associated with a poorer prognosis [9, 17]. In cases of well-differentiated tumors, (low-grade, Hyams grade I-II), the histological diagnosis is relatively straightforward. Higher-grade ONB tumors (Hyams grade III-IV) are less differentiated and can mimic other tumors, such as pituitary adenoma, melanoma, sinonasal endocrine carcinoma, lymphoma, or rhabdomyosarcoma [6, 15].

Future diagnostic approaches and subclassifications may incorporate molecular and genetic factors, such as DNA methylation patterns [16]. It is essential to distinguish ONB from other neuroendocrine tumors, since studies have demonstrated that non-ONB neuroendocrine tumors exhibit a higher propensity for metastatic spread [13]. The Hyams histological grading system is complemented by 2 clinical staging systems, the modified Kadish system [10, 11] and the TNM staging [18].

Low prevalence of ONB and absence of prospective studies have resulted in a lack of standardized therapeutic guidelines [6, 13]. The usual approach is a combination of tumoral resection and local radiotherapy [16], which typically results in recurrence-free rates of 60% to 100% [6]. Multiple surgical interventions may be required, as in this case. Traditionally, surgical resection is performed by a transcranial and transfacial approach with en bloc removal [16]. Recently, endoscopic resection has become more popular and has shown comparable results [16, 19]. Therapy should include neck dissection if nodal involvement is present [13, 16].

Studies indicate improved disease-free intervals and overall survival rates when postoperative radiation therapy is performed [16]. In this case, localized radiation therapy was recommended, and proton beam therapy was specifically advised to avoid harm of adjacent structures.

The role of chemotherapy in ONB remains controversial [13]. However, it was suggested for advanced disease or high Hyams grade tumors in addition to radiotherapy [16] and/or in palliative settings [6].

Recent studies have identified molecular pathways (eg, Sonic Hedgehog, VEGF, hTERT, TP53) and chromosomal instabilities as potential therapeutic targets [20-22]. Additionally, ONB shows similarities to SCLC at a molecular level in rodents [23]. Targeted therapies, including tyrosine kinase inhibitors, epidermal growth factor (EGFR) inhibitors, or immune checkpoint inhibitors, like programmed cell death ligand 1 (PD-L1), are under investigation but require further validation [16, 20].

Five-year overall survival ranges from 57% to 93% [6, 13] and primarily correlates with disease stage. Optimal surveillance protocols remain unclear. Since delayed recurrences occur [6], a prolonged follow-up is recommended [13].

In conclusion, paraneoplastic SIAD due to ONB is exceedingly rare but should be considered when no other cause of hyponatremia is identified. Overall, a comprehensive literature review of ONB as well as clear diagnostic and therapeutic guidelines are hindered by factors such as evolving or imprecise histological diagnoses, lack of randomized trials, limited follow-up data, and small case series due to the low prevalence of this disease. Further research is needed to provide more insight.

## Learning Points

- While rare, paraneoplastic SIAD can be caused by ONB and ONB should be ruled out, if no other cause is found, especially in SIAD suggesting a possible paraneoplastic cause (Type A).
- Primary therapy for ONB consists of surgical resection combined with radiotherapy.
- Because of the low prevalence of ONB, further research is needed to establish clear diagnostic and therapeutic guidelines.

## Data Availability

Data sharing is not applicable to this article as no datasets were generated or analyzed during the current study.

## Abbreviations

CT: computed tomography
MRI: magnetic resonance imaging
ONB: olfactory neuroblastoma
SCLC: small cell lung cancer
SIAD: syndrome of inappropriate antidiuresis
